# Tacrolimus dose requirement based on the CYP3A5 genotype in renal transplant patients

**DOI:** 10.18632/oncotarget.18150

**Published:** 2017-05-24

**Authors:** Lihui Qu, Yingying Lu, Meike Ying, Bingjue Li, Chunhua Weng, Zhoutao Xie, Ludan Liang, Chuan Lin, Xian Yang, Shi Feng, Yucheng Wang, Xiujin Shen, Qin Zhou, Ying Chen, Zhimin Chen, Jianyong Wu, Weiqiang Lin, Yi Shen, Jing Qin, Hang Xu, Feng Xu, Junwen Wang, Jianghua Chen, Hong Jiang, Hongfeng Huang

**Affiliations:** ^1^ Kidney Disease Center, The First Affiliated Hospital, College of Medicine, Zhejiang University, Hangzhou, China; ^2^ Kidney Disease Immunology Laboratory, The Third Grade Laboratory, State Administration Of Traditional Chinese Medicine Of PR China, Hangzhou, China; ^3^ Key Laboratory Of Multiple Organ Transplantation, Ministry Of Health, Hangzhou, China; ^4^ Key Laboratory Of Nephropathy, Zhejiang Province, Hangzhou, China; ^5^ Department of Biochemistry, LKS Faculty of Medicine, The University of Hong Kong, Hong Kong SAR, China; ^6^ Centre for Genomic Sciences, LKS Faculty of Medicine, The University of Hong Kong, Hong Kong SAR, China; ^7^ Shenzhen Institute of Research and Innovation, The University of Hong Kong, Shenzhen, Guangdong, China; ^8^ Department of Epidemiology, College of Medicine, Zhejiang University, Hangzhou, China; ^9^ Institute of Translational Medicine, College of Medicine, Zhejiang University, Hangzhou, China

**Keywords:** CYP3A5, FK506, renal transplantation, acute rejection

## Abstract

Tacrolimus (FK506) and cyclosporine A (CsA) are widely used to protect graft function after renal transplantation. The aim of the present study is to determine whether the single nucleotide polymorphism of CYP3A5 is a predictive index of FK506 dose requirement, and also the selection yardstick of FK506 or CsA treatment.We tested archival peripheral blood of 218 kidney recipients for CYP3A5 genotyping with PCR-SSP. Meanwhile, the dose of FK506 and CsA was recorded, blood concentration of the drugs was measured, and graft outcome was monitored.These results indicate that CYP3A5*AA/AG carriers need higher FK506 dose than CYP3A5*GG homozygote to achieve the target blood concentration. For CYP3A5*GG carriers, taking FK506 or CsA are both advisable. CYP3A5*AA/AG carriers preferred to CsA treatment depending on the graft outcomes and drug costs. CYP3A5 genotyping is a new approach to detecting FK506 dose requirement and a predictive index for the FK506 or CsA treatment selection in kidney recipients.

## INTRODUCTION

Tacrolimus (FK506) and cyclosporine A (CsA), two kinds of immunosuppressive drugs, have been widely used in the field of renal transplantation. As triple-immunosuppressive regimen, FK506-Mycophenolate Mofetil (MMF)- Prednisone (Pred) or CsA-MMF-Pred was applied mainly depended on doctors’ experience and drug economics. However, there was no any detail treatment guidelines for the drug selection.

FK506 could protect transplant function. Underdosing of immunosuppressive drugs could cause immunological graft rejection and influence the recipients’ survival quality. However, once the recipients accepted FK506 as their immunosuppressive drug, most of them would have to take it all life long. Furthermore, it could cause a series of side effects, such as toxicity to graft, infection, and even malignant tumors. Therapeutic FK506 monitoring has been carried out routinely, since target blood concentrations was expected to achieve with the lowest dose of drug given.

There were several reasons for the marked heterogeneity among individuals in blood concentrations of FK506, which were achieved by a standard body-weight-based dosing. According to recent studies, the variation was determined by genetic factors to some extent [1], and also manifested as the pharmacokinetic differences among ethnic groups [2]. MacPhee [3] reported that FK506 pharmacogenetics was associated with polymorphic expression of cytochrome P4503A5 (CYP3A5), an oxidative enzyme involved in FK506 absorption and metabolism. It was found that CYP3A5*A expressers require two-fold higher doses of FK506 than CYP3A5*G ones to achieve the target blood concentration [3].However, it was reported that CYP3A5 polymorphism could also affect CsA pharmacokinetics in living donor renal transplant recipients [4]

In this present study, we intend to determine CYP3A5*A and CYP3A5*G allele frequencies in Chinese kidney transplanted recipients and its association with FK506 or CsA dose requirement. We also intend to figure out whether the single nucleotide polymorphism of CYP3A5 could be a selection yardstick of FK506 or CsA treatment.

## RESULTS

### Demographic characteristics of patients

A cohort of 218 kidney recipients was recruited. The basic data of the patients were shown in Table 1. There were 123 patients accepting FK506 treatment and 95 patients accepting CsA treatment after transplantation. In FK506 treatment group, the frequency of CYP3A5*AA was 11/123, CYP3A5*AG 47/123, and CYP3A5*GG 65/123. In CsA treatment group, the frequency of CYP3A5*AA, CYP3A5*AG, CYP3A5*GG was 9/95, 34/95 and 52/95 respectively. 81 patients were included in the CYP3A5* AG group, not much less than the CYP3A5*GG group (137 patients), which is a different situation from it in white peoples and middle-eastern [1]. The age of CYP3A5*GG individuals in FK506 group were younger than that in CsA group (*p* = 0.0011, Table 1). There was no significant difference in gender, weight, HLA mismatch number or primary diseases between folks with different CYP3A5 genotypes or different therapy regime (Table 1).

**Table 1:** Demographic characteristics of patients

Characteristics	FK506		CsA	
	GG	AA/AG	GG	AA/AG
Patient, n (AA/AG)	65	58 (11/47)	52	43 (9/34)
Gender, M/F	45/20	32/26	33/19	30/13
Age, mean±sd, years	39.18±10.79^b^	39.24±10.34	46.10±11.41	41.56±11.48
Weight, mean±sd, kg	56.39±10.74	55.04±8.42	58.58±8.90	57.76±9.62
HLA mismatch, mean±sd	2.98±1.20	2.76±1.44	2.81±1.57	2.67±1.54
Cause of kidney disease, n				
Glomerular disease	59	51	48	40
Gouty nephropathy	1	1	0	0
Polycystic kidney disease	2	2	0	1
Others	3	4	4	2

^b^: *p* < 0.01 vs CYP3A5*GG with CsA treatment.

### FK506 dose adjusted concentration related to CYP3A5 genotype

**Figure 1:**
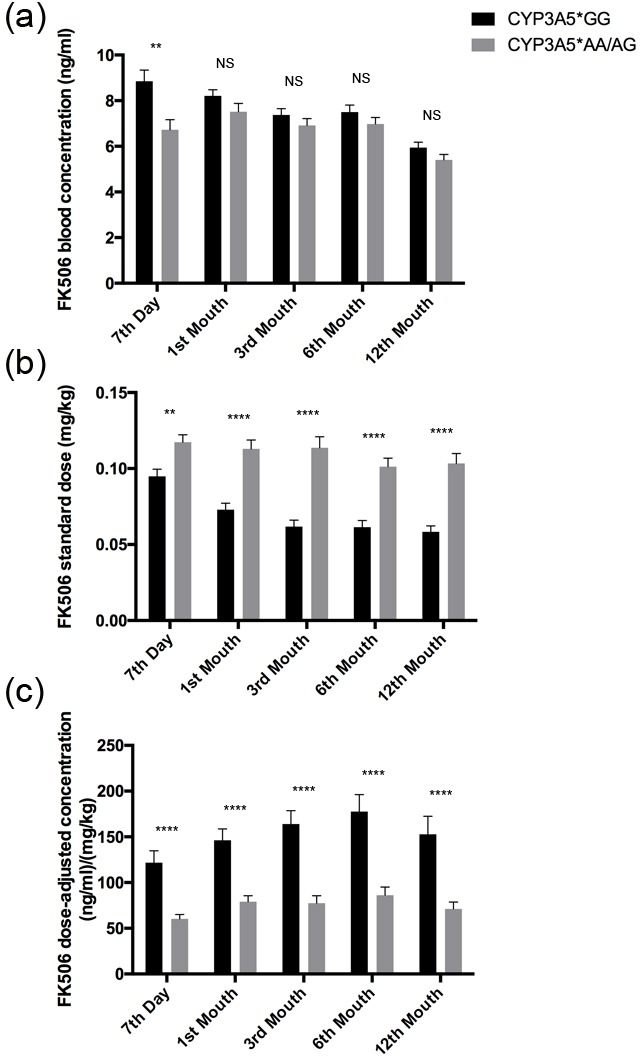
FK506 dose adjusted concentration related to CYP3A5 genotype FK506 blood concentrations **A.,** FK506 standardized dose **B.,** and dose normalized FK506 concentration **C.,** of CYP3A5* GG recipients and CYP3A5* AA/AG recipients at 7th day, 1^st^ month, 3^rd^ month, 6^th^ month and 12^th^ month after the kidney transplantation. NS: not significant, **: *p* < 0.01, ****: *p* < 0.0001. Error bars in graphs indicates SEM.

FK506 blood concentrations were measured on day 7, 1st month, 3rd month, 6th month and 12th month for each recipient after renal transplantation. CYP3A5*GG carriers taking FK506 treatment get the highest blood concentration (8.85±3.97 ng/ml) at day 7 post-transplantation with the standard dose(0.10±0.04mg/kg) (Figure 1a, 1b). They were then treated with a continuously decreased dose(Figure 1b), and consequently the FK506 blood concentration showed a gradual decrease to a stable level during the first year (Figure 1a). Regarding CYP3A5*AA/AG carriers, the blood concentration of FK506 was similar with CYP3A5*GG ones at all time points except for day 7 post-transplantation when dose adjustment was still in the early stage (*p* = 0.0012, Figure 1a). However, they consumed much more considerable dose of FK506 than CYP3A5*GG carriers to maintain an effective blood concentration at each time point (Figure 1b). Accordingly, the dose-adjusted concentration for CYP3A5*AA/AG was lower than that of CYP3A5*GG carriers at each time point during the first year (*p* < 0.0001, Figure 1c).

### CsA dose adjusted concentration related to CYP3A5 genotype

**Figure 2:**
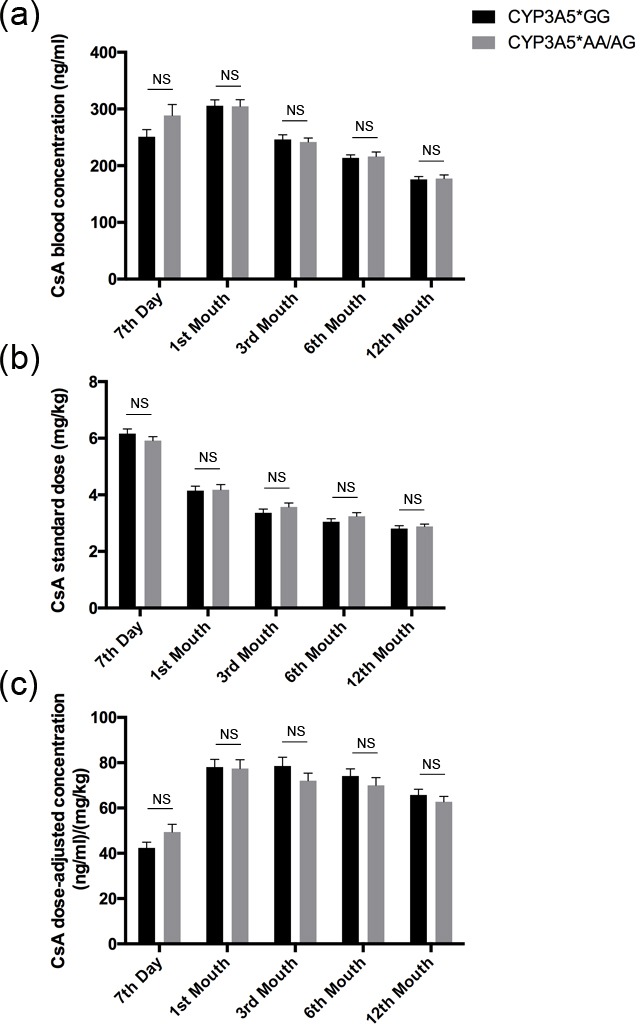
CsA dose adjusted concentration related to CYP3A5 genotype CsA blood concentrations **A.**, CsA standardized dose **B.** and dose normalized CsA concentration **C.** of CYP3A5* GG recipients and CYP3A5* AA/AG recipients at 7th day, 1^st^ month, 3^rd^ month, 6^th^ month and 12^th^ month after the kidney transplantation. NS: not significant. Error bars in graphs indicates SEM.

The influence of CYP3A5 genotype on dose-adjusted concentration was also analyzed for the patients who took CsA treatment. There was little if any difference in CsA blood concentration or CsA standard dose at any time point during the first year after kidney transplantation (Figure 2a, 2b). Consequently, no significant difference of CsA dose-adjusted concentration between CYP3A5*GG carriers and CYP3A5*AA/AG ones was found (Figure 2c).

### The relationship between CYP3A5 genotype and liver injury/renal function markers

**Figure 3:**
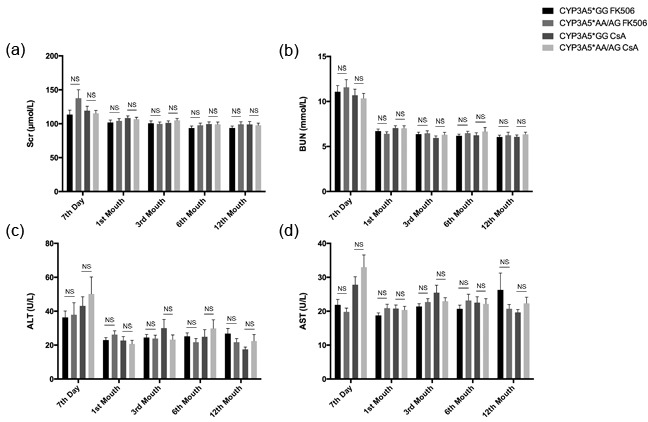
Clinical parameters of liver and kidney injury related to CYP3A5 genotype and different immunosuppressors Clinical parameters of kidney function, Scr **A.**, BUN **B.** and liver injury, ALT **C.**, AST **D.,** for each recipient was measured in 7 day, 1^st^ month, 3^rd^ month, 6^th^ month and 12^th^ month after the kidney transplantation. NS: not significant. Error bars in graphs indicates SEM.

The relationship between CYP3A5 genotype and liver/renal injury after kidney transplantation was analyzed for each patient. There were no significant differences in liver injury biomarkers alanine transaminase (ALT) or aspartate transaminase (AST), as well as renal function biomarkers serum creatinine (Scr) or bloodureanitrogen (BUN) in different CYP3A5 genotypes regardless of the therapy regime during the first year, as shown in Figure 3.

### The relationship between CYP3A5 genotype and acute rejection rate or graft loss rate or side effects

There were 218 patients in this cohort, and 21 of them had got acute rejection. For CYP3A5*GG carriers, those taking FK506 subjected to greater rejection rate than those taking CsA (6.15% vs. 19.23%, *P* = 0.030, Table 2). For CYP3A5*AA/AG patients, however, the acute rejection rate was similar between different treatment groups (8/58 vs. 6/43. *P* = 0.982, Table 2).

**Table 2:** Acute rejection rate in different CYP3A5 genotype

Characteristics	FK506		CsA	
	GG	AA/AG	GG	AA/AG
Patient, n (AA/AG)	65	58 (11/47)	52	43 (9/34)
Acute rejection, n(%)	4(6.15%)^a^	8(13.79%)	10(19.23%)	6(13.95%)
Acute rejection based on type of Immunosuppressive drugs, n(%)	12(9.76%)		16(16.84%)	
Total acute rejection, n(%)	28(12.84%)			

^a^ : *p* < 0.05 vs CYP3A5*GG with CsA treatment.

Univariate analysis showed that neither genotype no calcineurin inhibitor was associated with renal acute rejection. Induction therapy, however, influenced renal acute rejection rate. Multivariate Logistic regression analysis revealed similar results (Table 3). Importantly, there was a statistical difference of induction therapy rate between GG patients with FK506 treatment and GG patients with CsA treatment result (23.1% vs 57.7%, *p* < 0.0001).

**Table 3:** Logistic regression hazard ratios for renal acute rejection

	Univariate			Multivariate		
	RR	95%CI	*P*	RR	95%CI	*P*
Age	1.034	0.998-1.072	0.061	1.030	0.993-1.069	0.115
HLA mismatch	0.852	0.647-1.121	0.253	0.845	0.639 1.117	0.236
Genotype	1.184	0.535-2.619	0.677	1.212	0.529-2.777	0.650
Calcineurin inhibitor	1.873	0.840-4.178	0.125	1.419	0.604-3.334	0.422
Induction therapy	2.760	1.208-6.304	0.016	2.526	1.086-5.876	0.031

RR, relative risk; CI, con dence interval.

Among 95 initial CsA treated patients, 20 CYP3A5*GG carriers and 16 CYP3A5*AA/AG carriers shifted CsA to FK506 treatment after several years for some reasons. 7 CYP3A5*GG carriers shifted therapy regime when they encountered acute rejection, and never suffered second acute rejection. Another 2 CYP3A5*GG carriers, however, encountered acute rejection after therapy shift. The acute rejection rate didn't change much before or after shifting to FK506 treatment in CYP3A5*GG subgroup (7/20 vs. 2/20, *P* = 0.127, Table 4). In CYP3A5*AA/AG subgroup, it was because of acute rejection that 4 patients changed immunosuppressive therapy, and 1of them suffered second acute rejection after therapy shift. For CYP3A5*AA/AG subgroup, the acute rejection rate was also similar before and after treatment shift (4/16 vs. 2/16, *P* = 0.654, Table 4). There was no case in which FK506 was shifted to CsA in our study.

**Table 4:** Acute rejection rate before and after CsA shift to FK506

Genotype	GG			AA/AG		
Drug shift	non-shift	drug shift		non-shift	drug shift	
		before	after		before	after
Patient number (AA/AG)	32	20	20	27 (5/22)	16 (4/12)	16 (4/12)
Acute rejection n(%)	3(9.38%)	7(35%)	2(10.00%)	2(7.41%)	4(25%)	2(12.50%)

There was only one participant suffered graft loss in each group (CYP3A5*GG carriers with FK506 treatment, CYP3A5*GG carriers with CsA treatment from beginning to end, CYP3A5*AA/AG carriers with FK506 treatment, CYP3A5*AA/AG carriers with CsA treatment from beginning to end) during follow-up periods. There was no statistical significance in the graft survival rate among these groups (*P* = 0.304).

There was also no statistical significance in the the rates of side effects or postoperative complications, such as diarrhea (*p* = 0.371), drug-induced liver disease (DILD, *p* = 0.278), postoperative diabetes mellitus (PTDM, *p* = 0.079) and postoperative Leukocytopenia (*p* = 0.513) among these groups (Table 5).

**Table 5:** Side effects rate in different CYP3A5 genotype

	FK-GG (65)	FK-AA/AG (58)	CsA (non-shift)-GG (32)	CsA (non-shift)-AA/AG (27)	*P* value
Diarrhea	2	0	1	1	*p* = 0.371
DILD	0	0	0	1	*p* = 0.278
PTDM	1	2	4	3	*p* = 0.079
Postoperative Leukocytopenia	0	1	0	0	*p* = 0.513

PTDM: postoperative diabetes mellitus; DILD: Drug-induced liver disease.

### The relationship between CYP3A5 genotype and immunosuppressive therapy cost

**Figure 4:**
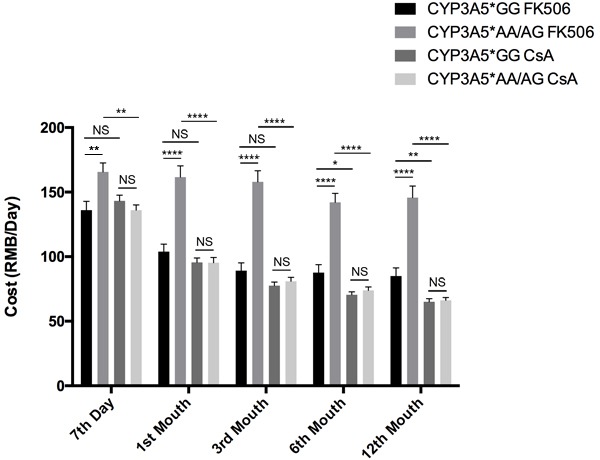
Costs of patients related to CYP3A5 genotype and different immunosuppressors Participants’ cost of immunosuppressive agents of different CYP3A5 genotypes. NS: not significant, *: *p* < 0.05, **: *p* < 0.01, ****: *p* < 0.0001, Error bars in graphs indicates SEM.

The cost of immunosuppressive agents of individual recipient was recorded at regular intervals. For patients with FK506 treatment, CYP3A5* AA/AG carriers cost much more than CYP3A5*GG carriers (Figure 4), since CYP3A5* AA/AG carriers need larger FK506 dose to maintain an effective blood concentration (Figure 1). For patients with CsA treatment, the cost of FK506 between the two genotypes was not statistically different at any time point. For CYP3A5* GG carriers, FK506 treatment showed similar expense with CsA treatment at the seventh day, first month and third month. However, in the 6th month and 12th month, CYP3A5* GG carriers treated with FK506 spend more than those treated with CsA. Meanwhile, for CYP3A5* AA/AG carriers, those treated with FK506 had to afford higher expense than those with CsA treatment (Figure 4).

## DISCUSSION

In our center, we had the standard triple-drug maintenance immunosuppressant included calcineurin inhibitor (FK506: trough level 5-10 ng/ml, or cyclosporine: trough level 200-300 ng/ml in half year after transplant), MMF or azathioprine, and Prednisone (in tapering doses from 80 to 10 mg/day) within the first month after transplantation for Chinese people [5–9]

It was reported that different ethnic groups require different doses of FK506 to achieve the target concentration [10–14]. The genotype showed that there was a slightly higher number of CYP3A5*GG than CYP3A5*AA/AG recipients, which was dramatically different from white people with very few CYP3A5*AA/AG recipients. Thus the ethnic could be one of the most important impact factors for the genotype.

Previous researches have suggested that the CYP3A5 genotype could predict the FK506 dose requirement to achieve target concentration in transplant recipients [15]. Evidently in our study, we also found that the CYP3A5*AA/AG recipients need more FK506 dose to achieve the target concentration than CYP3A5*GG recipients. In addition, the CYP3A5*GG group took shorter time to get the target therapeutic concentration and maintain a stable dose-adjusted concentration. We tried to check whether CYP3A5*AA/AG or CYP3A5*GG recipients could reach target blood concentration rapidly and efficiently when they were treated with lower CsA dose, like CYP3A5*GG recipients with lower FK506 dose treatment. However, it was hard for us to find a powerful support from this study.

On the other hand, we found some CYP3A5*GG carriers still needed higher FK506 doses. And some CYP3A5*AA/AG carriers just needed lower FK506 to keep the target blood concentration. Further research is necessary to explore the factors which induce the heterogeneity.

Previous studies also suggested the CYP3A5 genotype could be an important risk factor for the transplant rejection [16–18] and CYP3A5*AA/AG recipients had the higher rate to experience acute rejection [19, 20]. In the present study, however, the occurrence rate of acute rejection was similar between CYP3A5*GG recipients and CYP3A5*AA/AG recipients. For CYP3A5*GG patients, those treated with CsA suffered greater rejection rate than those taking FK506. The potential difference might have been exaggerated by the age difference between the two groups. What's more, the difference of induction therapy rate might account for the different acute rejection rate between these two groups, since induction therapy was risk factor for renal acute rejection (Table 3). More participants should be included in the cohort and age-paired analysis should be applied in the near future. For CYP3A5*AA/AG recipients, however, there was no statistical difference of acute rejection rate between different treatment group. Some other factors were reported to induce acute rejection, which remained to be identified though. In this study, we couldn't confirm the key causes of rejection like the HLA match (Table 3). That the cohort was not big enough might account for this.

In our study, there was no significant difference in the 3-year graft survival rate between different CYP3A5 genotypes (data not shown) regardless of the immunosuppressive agents regime, which was consistent to previous reports [21]. Short follow-up and small size could partly account for this.

The liver injury (ALT, AST) and kidney functions (Scr and BUN) exhibited no significant difference among the different genotype groups during the first year after the kidney transplantation. It seems that even high dose of FK506 taken by the CYP3A5*AA/AG groups did not cause serious burden on liver or kidney, as they were supervised strictly and judged by the professional doctors carefully.

It was accepted that FK506 was associated with lower rejection rates and longer graft life spans compared to CsA^22-26^, spending more however. In China, the average cost of tacrolimus in a stable renal-transplant recipient every day is approximately 1.8 times as high as CsA. In our study, for the FK506 treated patients, CYP3A5*GG carriers can sustain effective therapeutic concentration with lower FK506 dose and thus cost less than CYP3A5*AA/AG carriers. For the CsA treated patients, there was no significant difference of CsA expense between the two genotype groups. For CYP3A5*GG carriers, FK506 treatment had to afford higher expense than CsA treatment after half a year. For CYP3A5*AA/AG carriers, it cost even more to take FK506 treatment.

In conclusion, CYP3A5 polymorphism profoundly influenced pharmacokinetics of FK506 but not CsA. Taking all these into consideration, if a recipient is a CYP3A5*GG carrier, taking FK506 or CsA are both advisable. However, for a CYP3A5*AA/AG carrier, FK506 should not be the first choice due to the crushing costs and similar outcome. CYP3A5 genotyping is a reasonable approach to detecting FK506 dose requirement and a predictive index for FK506 or CsA treatment selection in kidney recipients.

## MATERIALS AND METHODS

### Patient population

The studies on human samples were approved by Ethics Committee of the First Affiliated Hospital, School of Medicine, Zhejiang University. And Experiments conducted was in accordance with Declaration of Helsinki. Written informed consent was obtained from all participants. 218 live-donor renal transplant recipients transplanted in our center between Jun. 2008 and Oct. 2010, and treated with FK506 or CsA, were invited to participate in the study. All the patients were treated with standard triple-drug maintenance immunosuppression included calcineurin inhibitor (FK506: trough level 5-10 ng/ml) or CsA, either MMF or azathioprine and prednisone which tapered doses from 80 to 10 mg/day within the first month after transplant. The FK506 and CsA dose requirement was assessed after transplantation. All demographic characteristics or clinical data of patients were blind to the researchers that were responsible for the drug blood concentration detection or CYP3A5 genotyping. All patients were followed up until Aug. 2014.

### Identification of CYP3A5 genotype

Blood samples of 2 to 3 milliliters were drawn from each patient in a vacuum tube containing ethylene-diaminetetracetic acid. Genomic DNA was extracted from 250μl whole blood by Blood Genomic DNA Miniprep kit (Axygen). Polymerase chain reaction using sequence-specific primers (PCR-SSP) was performed to identify CYP3A5 genotype (Human CYP3A5 genotyping PCR kit, Biosuper). 112μL reaction mixture consisting of 12μL genomic DNA, 0.8μL Taq DNA polymerase (Promega) and 100μL dNTP-Buffer that was provided in the kit was prepared and 10 μL reaction mixture was added into each well of the 96-well plates that have contained sequence-specific primers. Amplification involved 1 cycles of 96°C for 2 min, 5 cycles of 96°C for 20 seconds and 68°C for 60 seconds, 10 cycles of 96°C for 20 seconds,65°C for 50 seconds and 72°C for 45 seconds, 15 cycles of 96°C for 20 seconds, 62°C for 50 seconds and 72°C for 45 seconds followed by final extension at 72°C for 5 min. The PCR product was obtained and aliquots of 10μL were analyzed by electrophoresis on a 2.5% agarose gel containing ethidium bromide to check the sizes and amounts of the amplicons. Finally, the band patterns were visualized using an ultraviolet, and the CYP3A5 genotypes were identified according to the typing table.

### FK506 and CsA blood concentrations

The dose and blood concentrations of FK506 and CsA for recipients were measured at day 7, 1st month, 3rd month, 6th month and 12th month after renal transplantation, as well as hepatic and renal function. The FK506 blood concentration was measured using a PRO-Trac^TM^ II FK506 ELISA kit (DiaSorin Inc.), which were performed on a clinical analyzer.

### Statistics analysis

Mean and standard deviation (mean ± SD or mean ± SEM) were used to describe continuous variables, while percentage was used to describe categorical variables. Continuous variables were compared via analysis of variance (ANOVA). Comparison of frequencies was performed by chi-square test. Graft survival was analyzed with Kaplan- Meier method and differences between survival curves were calculated by the log-rank test. Logistic regression was conducted to reveal the influence factors of renal acute rejection. Values of *P* < 0.05 were considered statistically significant.
