# Correction: Tacrolimus dose requirement based on the CYP3A5 genotype in renal transplant patients

**DOI:** 10.18632/oncotarget.27561

**Published:** 2020-04-21

**Authors:** Lihui Qu, Yingying Lu, Meike Ying, Bingjue Li, Chunhua Weng, Zhoutao Xie, Ludan Liang, Chuan Lin, Xian Yang, Shi Feng, Yucheng Wang, Xiujin Shen, Qin Zhou, Ying Chen, Zhimin Chen, Jianyong Wu, Weiqiang Lin, Yi Shen, Jing Qin, Hang Xu, Feng Xu, Junwen Wang, Jianghua Chen, Hong Jiang, Hongfeng Huang

**Affiliations:** ^1^ Kidney Disease Center, The First Affiliated Hospital, College of Medicine, Zhejiang University, Hangzhou, China; ^2^ Kidney Disease Immunology Laboratory, The Third Grade Laboratory, State Administration of Traditional Chinese Medicine of PR China, Hangzhou, China; ^3^ Key Laboratory of Multiple Organ Transplantation, Ministry of Health, Hangzhou, China; ^4^ Key Laboratory of Nephropathy, Zhejiang Province, Hangzhou, China; ^5^ Department of Biochemistry, LKS Faculty of Medicine, The University of Hong Kong, Hong Kong SAR, China; ^6^ Centre for Genomic Sciences, LKS Faculty of Medicine, The University of Hong Kong, Hong Kong SAR, China; ^7^ Shenzhen Institute of Research and Innovation, The University of Hong Kong, Shenzhen, Guangdong, China; ^8^ Department of Epidemiology, College of Medicine, Zhejiang University, Hangzhou, China; ^9^ Institute of Translational Medicine, College of Medicine, Zhejiang University, Hangzhou, China

**This article has been corrected:** In Figures 1, 2, 3 and 4, the word ‘mouth’ was mistakenly used instead of ‘month’. The corrected Figures are shown below. The authors declare that these corrections do not change the results or conclusions of this paper.

Original article: Oncotarget. 2017; 8:81285–81294. 81285-81294. https://doi.org/10.18632/oncotarget.18150

**Figure 1:**
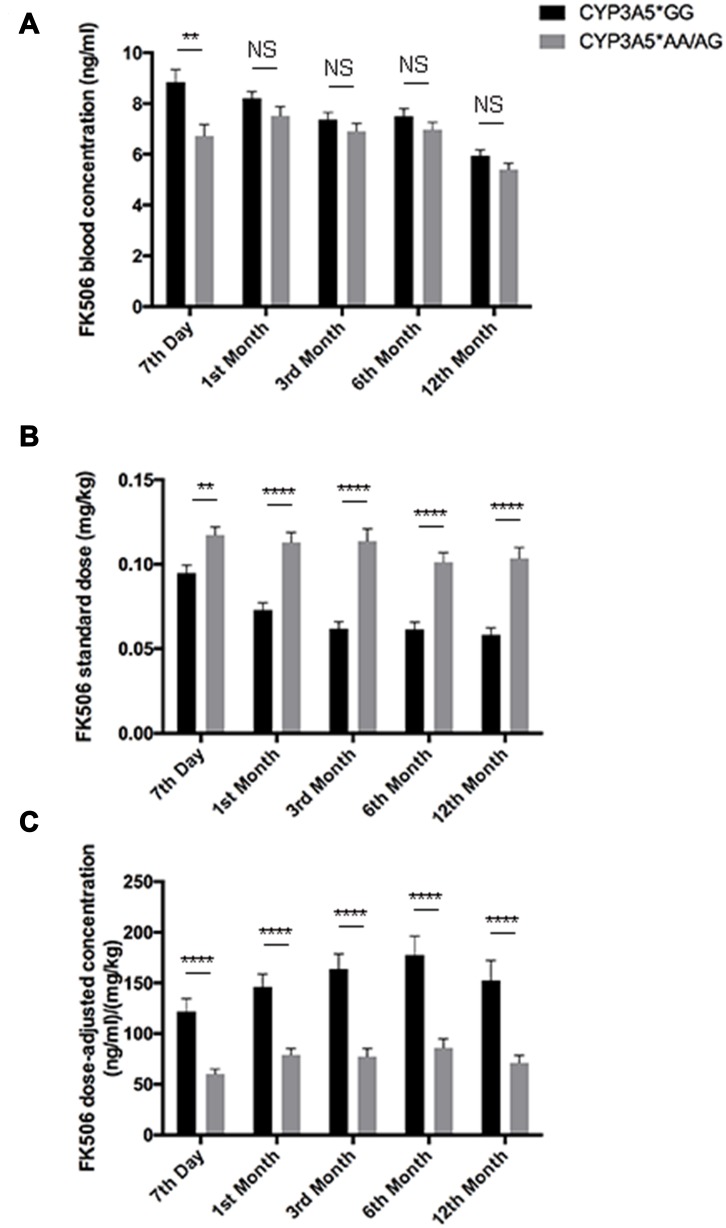
FK506 dose adjusted concentration related to CYP3A5 genotype. FK506 blood concentrations **A**., FK506 standardized dose **B**., and dose normalized FK506 concentration **C**., of CYP3A5* GG recipients and CYP3A5* AA/AG recipients at 7th day, 1^st^ month, 3^rd^ month, 6^th^ month and 12^th^ month after the kidney transplantation. NS: not significant, **: *p* < 0.01, ****: *p* < 0.0001. Error bars in graphs indicates SEM.

**Figure 2:**
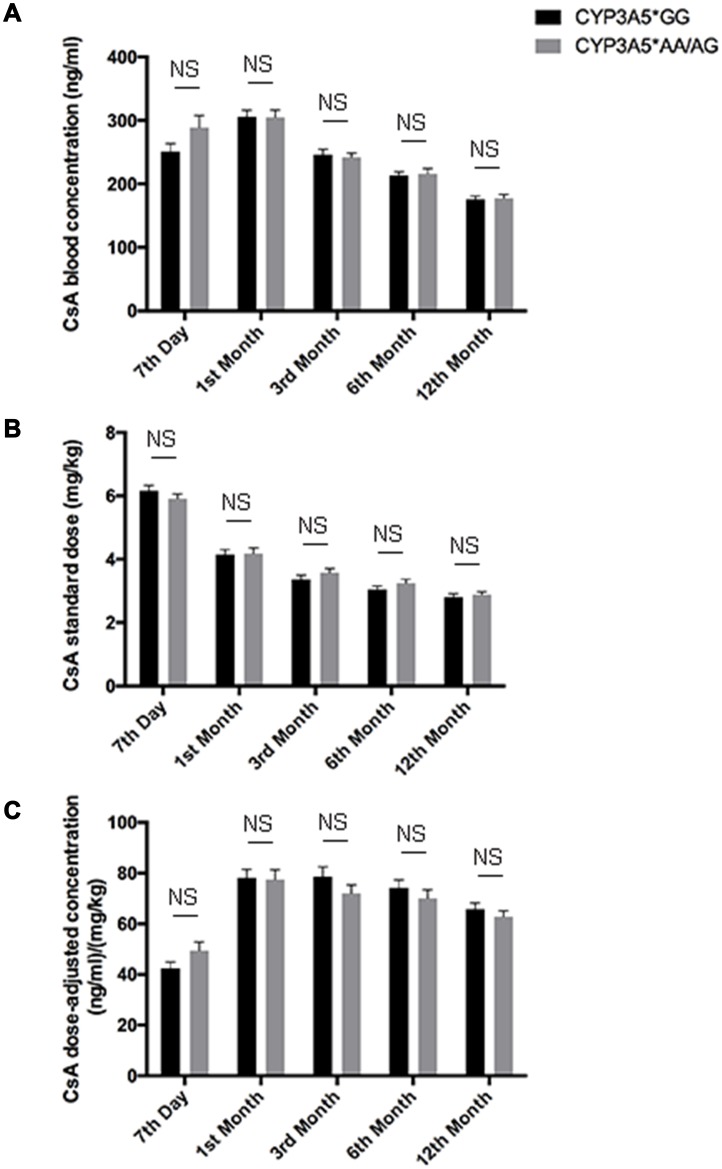
CsA dose adjusted concentration related to CYP3A5 genotype. CsA blood concentrations **A**., CsA standardized dose **B**. and dose normalized CsA concentration **C**. of CYP3A5* GG recipients and CYP3A5* AA/AG recipients at 7th day, 1^st^ month, 3^rd^ month, 6^th^ month and 12^th^ month after the kidney transplantation. NS: not significant. Error bars in graphs indicates SEM.

**Figure 3:**
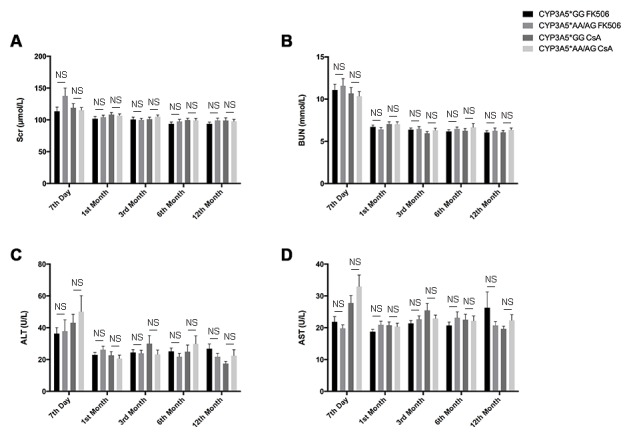
Clinical parameters of liver and kidney injury related to CYP3A5 genotype and different immunosuppressors. Clinical parameters of kidney function, Scr **A**., BUN **B**. and liver injury, ALT **C**., AST **D**., for each recipient was measured in 7 day, 1^st^ month, 3^rd^ month, 6^th^ month and 12^th^ month after the kidney transplantation. NS: not significant. Error bars in graphs indicates SEM.

**Figure 4:**
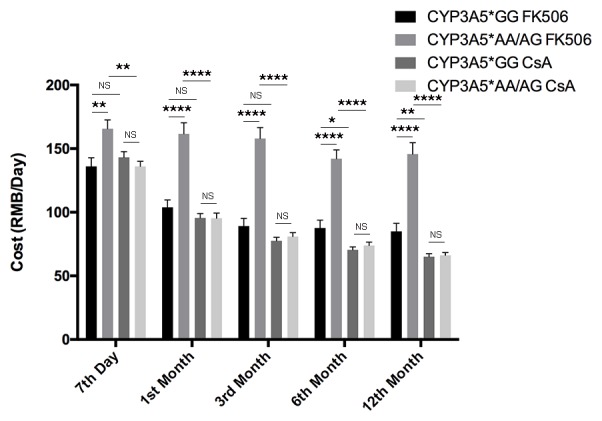
Costs of patients related to CYP3A5 genotype and different immunosuppressors. Participants’ cost of immunosuppressive agents of different CYP3A5 genotypes. NS: not significant, *: *p* < 0.05, **: *p* < 0.01, ****: *p* < 0.0001, Error bars in graphs indicates SEM.

